# The Relationship Between Organizational Culture and Organizational Commitment in Zahedan University of Medical Sciences

**DOI:** 10.5539/gjhs.v8n7p195

**Published:** 2015-12-15

**Authors:** Arbabisarjou Azizollah, Farhang Abolghasem, Dadgar Mohammad Amin

**Affiliations:** 1 Health Promotion Research Center, Zahedan University of Medical Sciences, Zahedan, Iran; 2 University of Sistan and Baluchestan, Zahedan, IR Iran; 3 Iranshahr School of Medical Sciences, Iranshahr, IR Iran

**Keywords:** organizational culture, organizational commitment

## Abstract

**Background and Objective:**

Organizations effort is to achieve a common goal. There are many constructs needed for organizations. Organizational culture and organizational commitment are special concepts in management. The objective of the current research is to study the relationship between organizational culture and organizational commitment among the personnel of Zahedan University of Medical Sciences.

**Materials and Methods:**

This is a descriptive- correlational study. The statistical population was whole tenured staff of Zahedan University of Medical Sciences that worked for this organization in 2012-2013. Random sampling method was used and 165 samples were chosen. Two standardized questionnaires of the organizational culture (Schein, 1984) and organizational commitment (Meyer & Allen, 2002) were applied. The face and construct validity of the questionnaires were approved by the lecturers of Management and experts. Reliability of questionnaires of the organizational culture and organizational commitment were 0.89 and 0.88 respectively, by Cronbach’s Alpha coefficient. All statistical calculations performed using Statistical Package for the Social Sciences version 21.0 (SPSS Inc., Chicago, IL, USA). The level of significance was set at P<0.05.

**Findings:**

The findings of the study showed that there was a significant relationship between organizational culture and organizational commitment (P value=0.027). Also, the results showed that there was a significant relation between organizational culture and affective commitment (P-value=0.009), organizational culture and continuance commitment (P-value=0.009), and organizational culture and normative commitment (P-value=0.009).

## 1. Introduction:

The idea to consider organizations as culture is a system of values shared among the members which is a relatively new phenomenon. Up to mid-1980s, the organizations were known as intellectual structures to coordinate and control the groups of people (Amin Mozaffari et al., 2008). The organizations had vertical hierarchy, departments, powerful relations and the staff. But organizations are beyond this definition as they have characters like human beings and might be flexible or lenient, unfriendly or supportive, innovative or conservative. Nowadays, the organizational theoreticians, approving the role that culture plays an important role in the organizational working life of its members and confirm this issue. Meanwhile, the origin of culture as an independent variable that affects the views and behaviors of the staff show that this concept originates from an institutionalization idea in more than 50 years ago (Robbins et al., 2013). Cultural attitudes influenced beliefs, behaviors and communication (Arbabisarjou, 2012). Culture affects the organizations in two ways. Of course approving the issue that culture has shared characteristics inside the organization does not mean that a culture cannot have several subcultures. In most of the big organizations, there are a dominant culture and some subcultures. The dominant culture indicates the main values which are shared among the majority of the members of the organization. When there is talk about the culture of an organization, the dominant culture of that organization is addressed. This macro-view is concerning culture that gives a different character to an organization. Subcultures are usually formed in big organizations and in reaction to the problems, situations or shared experiences that the members are faced with them. Subcultures are mainly created according to the sections of an organization and or geographical distances. For example, in the model presented by Cameron and Quinn (2005), four kinds of organizational culture were defined using the framework of competitive values:

1). Tribal culture (tribes, cooperatives): This culture finds flexibility a value and supervision and control over it is of less importance.
2). Adhocracy culture: This culture attaches high significance to the issues outside an organization, innovations, environmental developments, flexibility and open space for decision making.
3). Market-based culture: In this culture, supervision and control are more stressed on than flexibility, but external organizational issues and environmental changes are more important than the internal issues of an organization.
4). Hierarchical culture: This culture pays more attention to intra-organizational issues rather than the extra-organizational issues. Cameron and Quinn presented “instrument to assess the organizational culture” which is used to assess, evaluate and identify the relative superiority of four types of culture in the organization (Parvaneh, 2008).

However, nowadays the role of higher education centers and institutes is not hidden to anyone to materialize the objectives of the society as one of the main indexes of progress and distinction of different societies from each other. Universities as the major and key center to educate humans, have a special culture like the other organizations that could play a significant role in training qualified, expert people and entrepreneurs. This important issue requires the existence of suitable organizational culture in the state universities. These centers prepare the youth to make constant changes in the society as well as the world. Hence only attention and emphasis on technical and scientific qualifications are not noted at the universities, but the university feels a huge cultural responsibility on its burden as a cultural and culture-making institution. Organizational theoreticians believe that culture firstly determines the organizational border, secondly injects a type of feeling of identity into the body of the members of the organization and thirdly creates a kind of commitment in the people toward something which is more than the personal interests of an individual. In other words, culture is considered as a control factor which causes formation of the views and behavior of the staff through presentation of suitable criteria that might either push the organization forward or stop it from moving (Pouramen, 2001).

A strong organizational culture forms the staff’s behavior, coordinates their treatment, creates shared beliefs, work commitment, organizational identity for the staff, specifies the way the individuals look, prevents disorder in an organization, reduces the external control (creates self-control) and reduces contradiction, costs and job dissatisfaction (Moshabaki & Rahmani, 2009).

Institutionalization of an organization causes the staff and members of the organization to find a shared understanding of what is suitable and pleasant (toward a type of targeted behavior in principle). That is why when an organization changes to an institution, certain models of behavior are accepted by all the members of the organization and these behavioral samples are seen everywhere in the organization. As it is noticed, this is the same role played by the culture of the organization. Thus understanding of what the organizational culture makes helps the method to create and continue it so that one could better judge and anticipate the behavior of the individuals in the organization (Robbins et al., 2013). Dale and Kennedy, 1982 believe that culture is the most important effective factor on success or failure of an organization. They define four dimensions for the culture:

1). Values, 2). Epical heroes, 3). Religious rituals and method of worship, 4). Communication network of culture (Cameron & Quinn, 2005).

Considering the importance of the impact of the organizational culture on organization, the directors should always look for ways to identify, change and develop organizational culture so that they could affect the individual behavior and level of organization and facilitate access to the objectives of the organization for themselves and others (Moshabaki & Rahmani, 2009).

Thus, it could be said that the management of organizational culture has to know and use the existing culture optimally, to change and weaken the beliefs, values and the norms of the demand and to stabilize the pleasant culture. Therefore, the concept of culture makes sense when the vast changes in the organization are managed. The change of the organization is not just the change of structure, but it is the change in the culture of the organization. An attempt to change the culture of organization, when there is no correct understanding of the power of culture and its role in the organization leads to failure most of the time and this issue make many current strategic planners have a special emphasis on identifying the principal values of the organizations.

But it should be said that although the organizational culture is under the influence of bigger systems that surround it, the organizational culture of each set is the product of the structure, social, political and economic relations of every society (Vipul, Benyoucef, & Deshmukh, 2007). However in the current era, the culture of educational organizations face major challenges and most of these challenges lead to restructuring, re-engineering and minimizing their size. The current environment of the organizations has become complicated and difficult and this has caused some problems in this regard for their leaders. This issue requires the high flexibility of the leaders to remove the problems and confront the fluctuating environment around the organizations. It is noticed in some organizations that the infra-structural beliefs, values and hypotheses of the members of the organization and its leaders are not in the same direction which causes problems in the staff’s commitments toward the organization and subsequently leads to its low performance and absence and departure of the staff.

Organizational commitment is a stabilizing force that binds individuals to organizations (Bentein, Vandenberg, Vandenberghe, & Stinglhamber, 2005; Meyer & Herscovitch, 2001). Organizational commitment is one of the most commonly examined attitudes in the organizational sciences literature (see Meyer et al., 2002 for a quantitative review) and has particularly interested researchers since Allen and Meyer (1990) proposed a three-dimension model of the construct. The concept of organizational commitment is central to organizational behavior research. Organizational commitment is defined as an individual’s attitude towards an organization that consists of (a) a strong belief in, and acceptance of, the organization’s goals and values; (b) a willingness to exert considerable effort on behalf of the organization; and (c) a strong desire to maintain membership in the organization (Mowday et al., 1982). Organizational commitment has three primary components: (1) a strong belief in and acceptance of the organization’s goals and values; (2) a willingness to exert considerable effort on behalf of the organization; and (3) a strong desire to remain with the organization (Porter et al., 1974). Highly committed employees intend to stay within the organization and to work hard toward its goals (Luthans, McCaul, & Dodd, 1985). Meyer and Allen (1991) argued that there were three types of organizational commitment: (1) Affective Commitment: refers to the employee’s emotional attachment to, identification with, and involvement with the organization. Employees with a strong affective commitment continue employment with the organization because they want to do so. (2) Continuance Commitment refers to an awareness of the costs associated with leaving the organization. Employees whose primary link to the organization is based on continuance commitment remain because they need to do so. (3) Normative Commitment reflects a feeling of obligation to continue employment. Employees with a high level of normative commitment feel that they ought to remain with the organization. Career identity can be conceptually tied to work commitment (e.g. Dubin & Champoux, 1975), organizational commitment (Salancik, 1977) and organizational citizenship (Organ & Ryan, 1995). Thus, career motivation may positively correlate with organizational commitment. Although affective, continuance, and normative commitment are used to capture the multidimensional nature of organizational commitment, affective commitment is considered a more effective measurement of organizational commitment. Employees with strong affective commitment would be motivated to higher levels of performance and make more meaningful contributions than employees who expressed continuance or normative commitment (Brown, 2003. Thus, affective commitment alone is one of the key concepts of employee behavior. In this study we examined the association between career motivation and affective organizational commitment

Despite the importance of culture and its effect on organizational commitment of educational organizations, unfortunately the suitable bed for its growth is not available in our country and a ground for more growth should be provided to materialize this issue. But it seems that the University of Medical Sciences could pave the ground for creating commitment of the university and other service institutions to follow the duty to support work and noble capital and to pursue growth and improvement of the organizational commitment and issues as far as the organizational culture is concerned. Also a more useful performance should be predicted to ultimately achieve the goals of 2025 perspective. Meanwhile the researcher intended to study the relationship between organizational culture and organizational commitment at Zahedan University of Medical Sciences.

## 2. Materials & Methods

This is a descriptive–correctional study. The statistical population comprised of all personnel working at Zahedan University of Medical Sciences in 2012-2013. Random sampling method was used and 165 samples were selected. Two standardized questionnaires of the organizational culture (Edgar Shine, 2003) and organizational commitment (Meyer & Allen, 2002) were used. The face and construct validity of the questionnaires were approved by the lecturers of management and experts. Reliability of questionnaires of the organizational culture and organizational commitment were 0.89 and 0.88 respectively, by Cronbach’s Alpha coefficient. They consisted of 43 and 24-item closed items. Questionnaire about organizational commitment was three sub-scale (affective, normative, continuance). It used a 5-point Likert scale ranging from 1 (strongly disagree) to 5 (strongly agree). The data entered computer. All statistical calculation performed using Statistical Package for the Social Sciences version 21.0 (SPSS Inc., Chicago, IL, USA). The level of significance was set at P<0.05.

## 3. Findings

According to Table 1, the finding showed that organizational culture variable has the highest average (3.71) and the continuance commitment variable the lowest average among the variables of the study (3.25). But in general, the average of the variables of the study did not have significant differences with one another.

**Table 1:** Central indexes and dispersion of the variables of the study

Variables	Criterion deviation	Mean	Average	N
organizational commitment	**0.752**	3.4	3.38	**165**
continuance commitment	**0.857**	3.3	3.25	**165**
affective commitment	**0.794**	3.4	3.28	**165**
normative commitment	**0.731**	3.4	3.3	**165**
Organizational culture	**0.455**	3.8	3.71	**165**

**Test of Hypotheses:**

There is a significant relation between the organizational culture and the organizational commitment.

According to the results of Table 2, there was a significant and positive correlation between organizational culture and organizational commitment, thus the hypothesis of our study was confirmed that there is a significant relation between organizational culture and organizational commitment.

**Table 2:** Correlation co-efficient associated with organizational culture and organizational commitment

Variable	N	R Pearson Correlation	Level of significance
Organizational culture and organizational commitment	165	0.419	0.027

There is a significant relation between organizational culture and affective commitment.

Conducting the relevant test at certainty level of 95%, the P-value was 0.009 and thus the zero hypothesis was rejected, i.e., there is a significant relation between organizational culture and affective commitment, and considering the positive correlation coefficient mark, it could be said that this relation is of positive type. Also considering the amount of 0.52, it should be noted that the relation between the two variables is an average upward relation. Thus we conclude that the more the organizational culture increases, a certain amount could consequently lead to affective commitment by the individuals of that organization (Table 3).

**Table 3:** Correlation test for the most important hypothesis (α = 0.05, *N* = 165)

		Affective commitment.	Organizational culture
Organizational culture	Pearson correlative coefficient	0.52	1
	Level of significance (bilateral)	0.009	-
Affective commitment	Pearson correlative coefficient	1	0.52
	Level of significance (bilateral)	-	0.009

There is a significant relation between organizational culture and continuance commitment.

Conducting the correlation test at certainty level of 95%, the P-value was 0.009 and this shows that there is a significant relation between organizational culture and continuance commitment. Considering the positive correlation coefficient mark, it could be said that this relation is of positive type. Also considering the amount of 0.397, it said that the relationship between the two variables is an average upward relation (table 4). Thus it concluded that the more the organizational culture under study becomes operational, a certain amount could consequently lead in continuance commitment by the individuals of that organization.

**Table 4:** Correlation test for hypothesis of organizational culture and continuance commitment (α = 0.05, *N* = 165)

		Continuance commitment	Organizational culture
Organizational culture	Pearson correlative coefficient	0.397	1
	Level of significance (bilateral)	0.009	_
Continuance commitment	Pearson correlative coefficient	1	0.397
	Level of significance (bilateral)	_	0.009

There is a significant relation between organizational culture and normative commitment.

Conducting the correlation test at certainty level of 95%, the P-value was 0.003 and this showed that there was a significant relationship between organizational culture and normative commitment, and considering the positive correlation coefficient mark. It could be said that this relationship is positive type. Also considering the amount of 0.513, it shows that the relation between the two variables is an upward relation (Table 5).

**Table 5:** Correlation test for hypothesis of organizational culture and normative commitment (α = 0.05, *N* = 165)

		Normative commitment	Organizational culture
Organizational culture	Pearson correlative coefficient	0.513	1
	Level of significance (bilateral)	0.003	_
Continuance commitment	Pearson correlative coefficient	1	0.513
	Level of significance (bilateral)	_	0.003

## 4. Conclusion

As it has shown in the results, conducting the test of the main hypothesis at certainty level of 95%, P-value= 0.027 was obtained and therefore, the zero hypothesis was rejected (Table 2). It means that there was a significant relationship between the organizational culture and the organizational commitment, and considering the positive correlation coefficient mark, it could be said that this relationship is positive type. Also considering the amount of 0.411, it should be noted that the relation between the two variables is an average upward relationship (table 2). Thus it concluded that the more the organizational culture increases, a certain amount could consequently lead to organizational commitment by the tenured staff of the university. Hill (2003) conducted a study of eight organizations in South African countries, referred to organizational structure, reward system, organization appraisal, organizational commitment, organization management and organizational culture as the effective factors on the organizational development and growth. Also, in the hypothesis about the relation between organizational culture and affective commitment, the result was (0.52) at certainty level of 95% showing P-value=0.009 (Table 3). It showed a positive relation, thus it concluded that the more affective commitment exists in the organization, a certain amount could consequently lead to increase and growth of organizational culture by the individuals associated with that organization. The outcomes of Raynor (2008) showed in a research that entrepreneurship strategies of the organization are particularly in line with attracting the consent of the customers of the organization and human resources development, and has a positive relation with development of the organizational culture (Raynor, 2007). Also in the outcomes obtained from the hypothesis of the relation between organizational culture and continuance commitment, conducting correlation test, the result of 0.397 at the certainty level of 95% and P-value= 0.009 was obtained. It showed that there is a significant relation between the organizational culture and the continuance commitment, and considering the positive correlation coefficient mark, it could be said that this relation was of positive type since the relation between the two variables was an average upward relation. Hence we conclude that the more the continuance commitment are made operational in the society, a certain amount could consequently lead to growth and improvement of the organizational culture by the individuals of that organization. The outcomes of the hypothesis showed that there is a positive relation between organizational culture and normative commitment. Conducting the correlation test at certainty level of 95%, the P-value= 0.003 was obtained. It showed that there is a significant relation between organizational culture and normative commitment, and considering the positive correlation coefficient mark, it could be said that this relation is of positive type. Considering the quantity of 0.683, it should be said that the relation between the two variables is an upward relation. The outcomes of the study are in line with the studies conducted by (Raynor, 2007; Vipul et al., 2007; Furst, 2005; Baqerzadeh, Ezatollah, & Moafi Madani, 2009) and (Javanmardi, Seyed Mahmoud, Mehdi, & Khoobshani, 2011).

Organizational commitment refers to identification with and loyalty to the organization and its goals (Blau & Boal, 1987) which Mowday et al. (1982) defined as the relative strength of an individual’s identification with and involvement in a particular organization. In particular, commitment is characterized by three factors: a strong belief in and an acceptance of the organization’s goals and values; a willingness to exert considerable effort on behalf of the organization; and a strong desire to maintain membership in the organization. It has been found that organizational commitment is positively related to organizational culture and job satisfaction of hospital nurses (Blegen, 1993; Al-Aameri, 2000).

Therefore, enhancing professional commitment in staff has the potential to produce benefits for both the individual and their organization (Cohen, 1998, 1999).

Therefore commitment in the staff following the culture governing the organizations and this causes self-confidence and feeling of value in the staff and also paves the ground for commitment in the organization with a self-management approach. Educational organizations could use the existing achievements in line with their interests like the other production institutes. Hence the policy-makers and theoreticians of the educational institutes and universities should pave the ground for the development of the beliefs and the growth of the governing values and talents affiliated with them instead of restricting the staff. They should support the staff financially and spiritually to achieve the desirable outcomes as far as the organizational commitment is concerned. However the outcomes of the current study show that using and propagating an atmosphere full of behaviors, norms, beliefs and positive approaches in an organization could provide the most optimal working environment. But there is a long way ahead to make the changes related to the organizational culture and in order to make the organization dynamic through creation, growth and applied nature of growth and development, the commitment of the university could be considered as the fundamental priority that has to grow in each and every one of the tenured staff of the university.

**Ethical Considerations**

Ethical considerations of the study contained applied methods and tools, the aim of study, obtaining verbally informed consent, confidentiality of information, and withdrawal from the study at will. The participants in this research provided oral informed consent and a check mark in questionnaire taken their agreement for participation.

This research is a part of a MA thesis in educational administration which had approved in Research Council of Faculty of Educational Sciences and Psychology affiliated to University of Sistan and Balouchestan, Iran.
